# Sustainable bioenergy production with little carbon debt in the Loess Plateau of China

**DOI:** 10.1186/s13068-016-0586-y

**Published:** 2016-08-02

**Authors:** Wei Liu, Cheng Peng, Zhifen Chen, Yue Liu, Juan Yan, Jianqiang Li, Tao Sang

**Affiliations:** 1 State Key Laboratory of Systematic and Evolutionary Botany, Institute of Botany, Chinese Academy of Sciences, Beijing, 100093 China; 2 Beijing Research Center for Information Technology in Agriculture, Beijing Academy of Agriculture and Forestry Sciences, Beijing, 100097 China; 3 China Academy of Urban Planning and Design, Beijing, 100044 China; 4 Institute of Geographical Sciences and Natural Resources Research, Chinese Academy of Sciences, Beijing, 100101 China; 5 Key Laboratory of Plant Germplasm Enhancement and Speciality Agriculture, Wuhan Botanical Garden, Chinese Academy of Sciences, Wuhan, 430074 Hubei China; 6 Key Laboratory of Plant Resources and Beijing Botanical Garden, Institute of Botany, Chinese Academy of Sciences, Beijing, 100093 China

**Keywords:** Bioenergy, Carbon debt, Climate change, Land-use change, Marginal land, *Miscanthuslutarioriparius*

## Abstract

**Background:**

As a key strategy for mitigating global climate change, bioenergy production by reducing CO_2_ emissions plays an important role in ensuring sustainable development. However, land-use change by converting natural ecosystems into energy crop field could create a carbon debt at the beginning. Thus, the potential carbon debt calculation is necessary for determining a promising bioenergy crop production, especially in the region rich of marginal land.

**Results:**

Here, we used high-resolution historical land-use data to identify the marginal land available and to evaluate the carbon debt of planting *Miscanthus* in the Loess Plateau, China. We found that there were 27.6 Mha for energy production and 9.7 Mha for ecological restoration, with total annual production of 0.41 billion tons of biomass. We also found that soil carbon sequestration and total CO_2_ mitigation were 9.3 Mt C year^−1^ and 542 Mt year^−1^, respectively. More importantly, the result showed that planting *Miscanthus* on marginal land in the Loess Plateau only took 0.97 years on average to repay the carbon debt.

**Conclusions:**

Our study demonstrated that *Miscanthus* production in suitable marginal land in the Loess Plateau can offer considerable renewable energy and mitigate climate change with little carbon debt. These results suggested that bioenergy production in the similar arid and semiarid region worldwide would contribute to carbon sequestration in the context of rapid climate change.

**Electronic supplementary material:**

The online version of this article (doi:10.1186/s13068-016-0586-y) contains supplementary material, which is available to authorized users.

## Background

With increased global demand for renewable energy and response to climate change, bioenergy was suggested to be an important energy source that might substitute fossil energy in the near future [1, 2]. Reducing CO_2_ emissions and mitigating climate change are one of the important roles of bioenergy in ensuring sustainable development. Previous studies showed that land-use change (LUC) associated with biofuel feedstock production, especially with the conversion of natural ecosystems into energy crop field, could create a carbon debt at the beginning [3–7]. Recently, people paid close attention to this environmental cost of broad-scale bioenergy production [8–13]. One reasonable solution is to grow the second-generation energy crops on marginal land, taking advantages from both strong carbon sequestration ability of the perennial crops and little carbon debt associated with LUC [14–16]. Toward this direction, candidates of production locations and second-generation energy crops have been proposed and studied [17–19].

The Loess Plateau in China has the vast areas of marginal land and holds a great potential for the production of perennial herbaceous energy crops [20, 21]. As a cradle of the ancient Chinese civilization, it has been used for grain production for more than 2000 years. Most of natural land, e.g., forests, shrublands, or grasslands, was converted into arable land with population expansion, even cultivation on steep slopes [22]. Irrational land use created major environmental problems, such as soil erosion, which turned this region into one of the most eroded zones of the world [23]. Moreover, these conflicts between human and environment increased with the rapid social-economic development in the recent 30 years [24]. As the main driving force for LUC [25], bioenergy production in large scale may bring sustainable solution to this region.

*Miscanthus* is a C4 plant capable of maintaining high-photosynthetic rates and producing high biomass in the cool climate [15, 26, 27]. As a perennial grass with high water and nutrient use efficiencies, *Miscanthus* can be grown on marginal land without heavy irrigation or fertilization [28–30]. Due to its high cellulose content [31], *Miscanthus* is considered to be a promising second-generation energy crop to domesticate and cultivate in the north China [21]. Previous studies demonstrated that *Miscanthuslutarioriparius*, an endemic species in central China, was able to adapt to the semiarid regions and produced high biomass in the Loess Plateau [29, 32]. Furthermore, growing this high-biomass plant in the Loess Plateau had positive environmental impacts, which help to retain deep-soil water and hold a great potential for carbon sequestration and soil restoration in this heavily soil-eroded region [32–34].

However, it remains unclear about the potential carbon debt of converting vast areas of marginal land in large scale into *Miscanthus* field in the Loess Plateau. In this study, we proposed a new approach to identify suitable marginal land using recent 30-year land-use data set with high-spatial resolution of 30 × 30 m in the Loess Plateau. We then evaluated the yield potential using the previous yield model under three climatic scenarios in the 21st century and the carbon debt based on the biomass and the rate of soil carbon stock accumulation from the 4-year *Miscanthus* field. Our work demonstrated that the Loess Plateau is an ideal region for developing bioenergy crops.

## Results and discussion

We calculated the areas of six land-use change (LUC) types from 1980 to 1990, from 1990 to 2000, and from 2000 to 2008, including from cropland to grassland, from cropland to woodland, restoration from sandy and saline, vegetation change, conversion into cropland, and conversion into sandy and saline land (Additional file 1: Table S1). We also calculated the spatial density distribution of these LUC types (Additional file 1: Figure S1). These results indicated that the Loess Plateau underwent rapid land-use change in the recent 30 years.

**Fig. 1:**
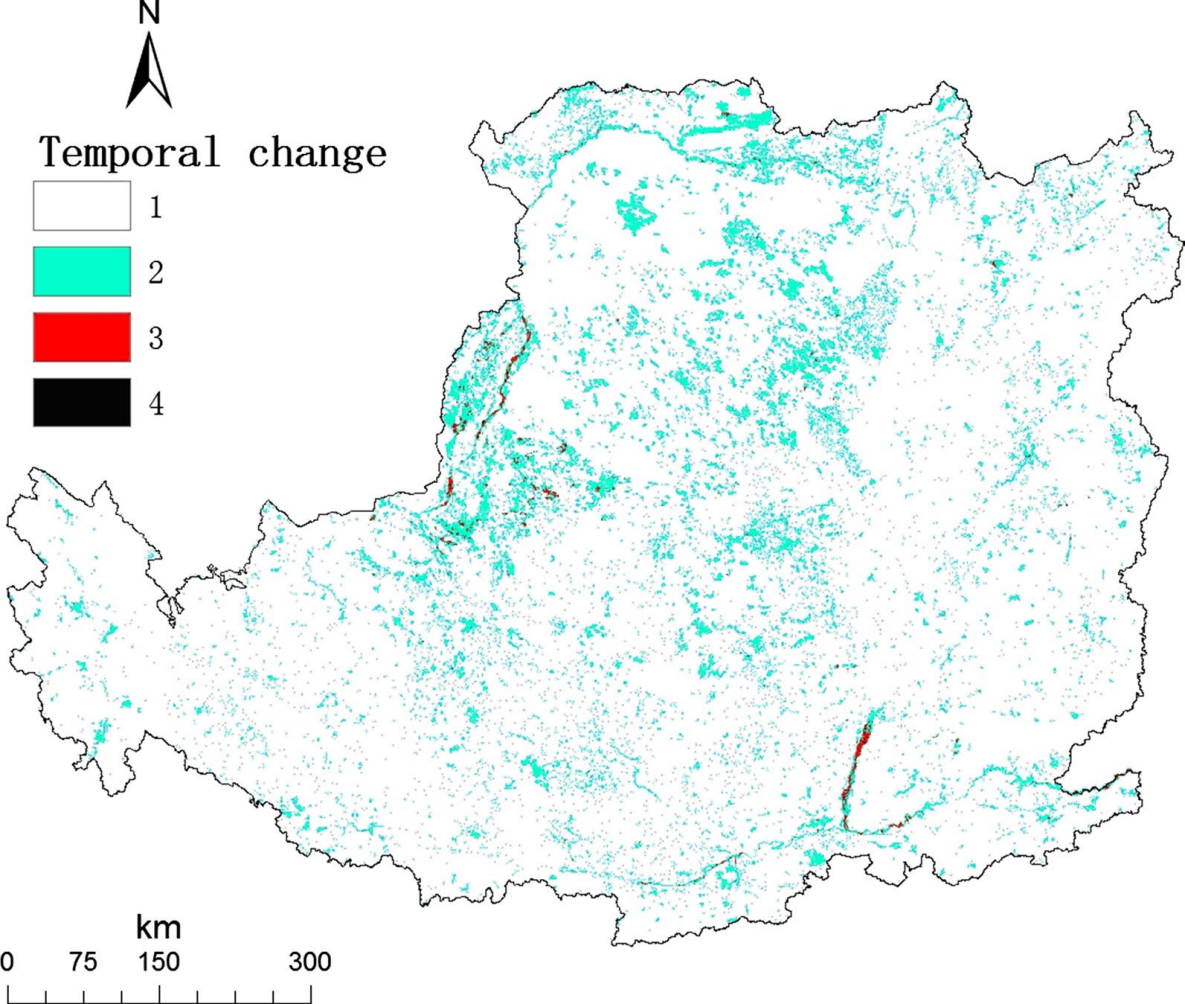
Map of the temporal change of land use in the Loess Plateau. For a given grid cell, its temporal change from 1980 to 2008 was calculated using the number of land-use types in 4 years. The value of temporal change ranged from 1 to 4

**Fig. 2:**
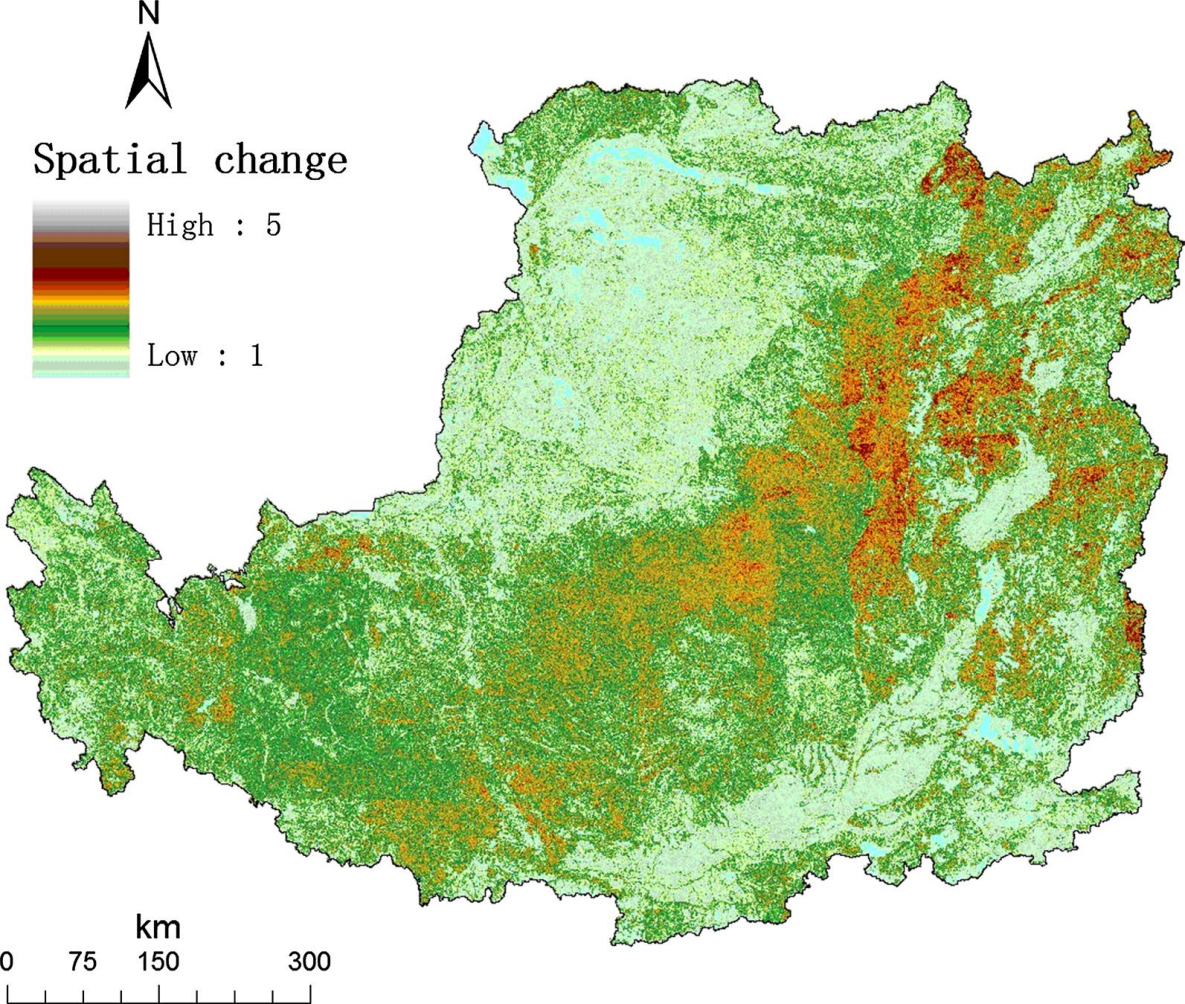
Map of the average spatial change of land use in the Loess Plateau

**Fig. 3:**
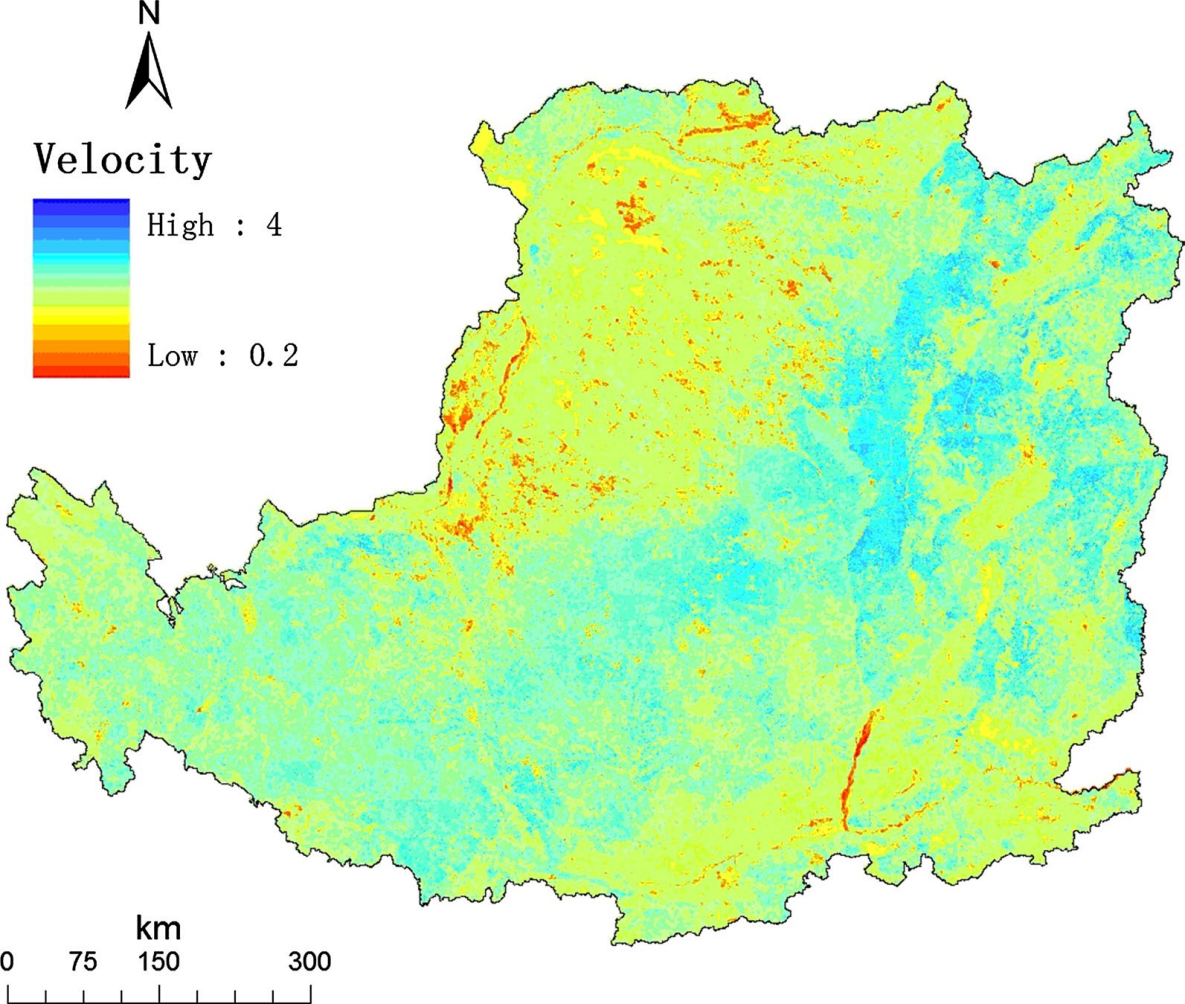
Map of the velocity of land-use change in the Loess Plateau

We further obtained the distribution of the temporal change (Fig. 1) and the distribution of the average spatial change from 1980 to 2008 in the Loess Plateau (Fig. 2). We calculated the velocity of LUC, as the ratio of the temporal change and the spatial change at a given grid. The distribution of the velocity of LUC 1980 to 2008 in the Loess Plateau was shown in Fig. 3.

A grid with frequent land-use change over time is not suitable for agriculture, pasture, or forest due to rapid land-use change in the Loess Plateau in the recent 30 years (Additional file 1: Table S1 and Figure S1). At the same time, a grid with relatively spatially homogeneous is a suitable candidate for marginal land to be converted to *Miscanthus* field, because bioenergy production needs vast area in large scale. In the sense, the area with high velocity of land-use change is indicative of ideal marginal land.

**Fig. 4:**
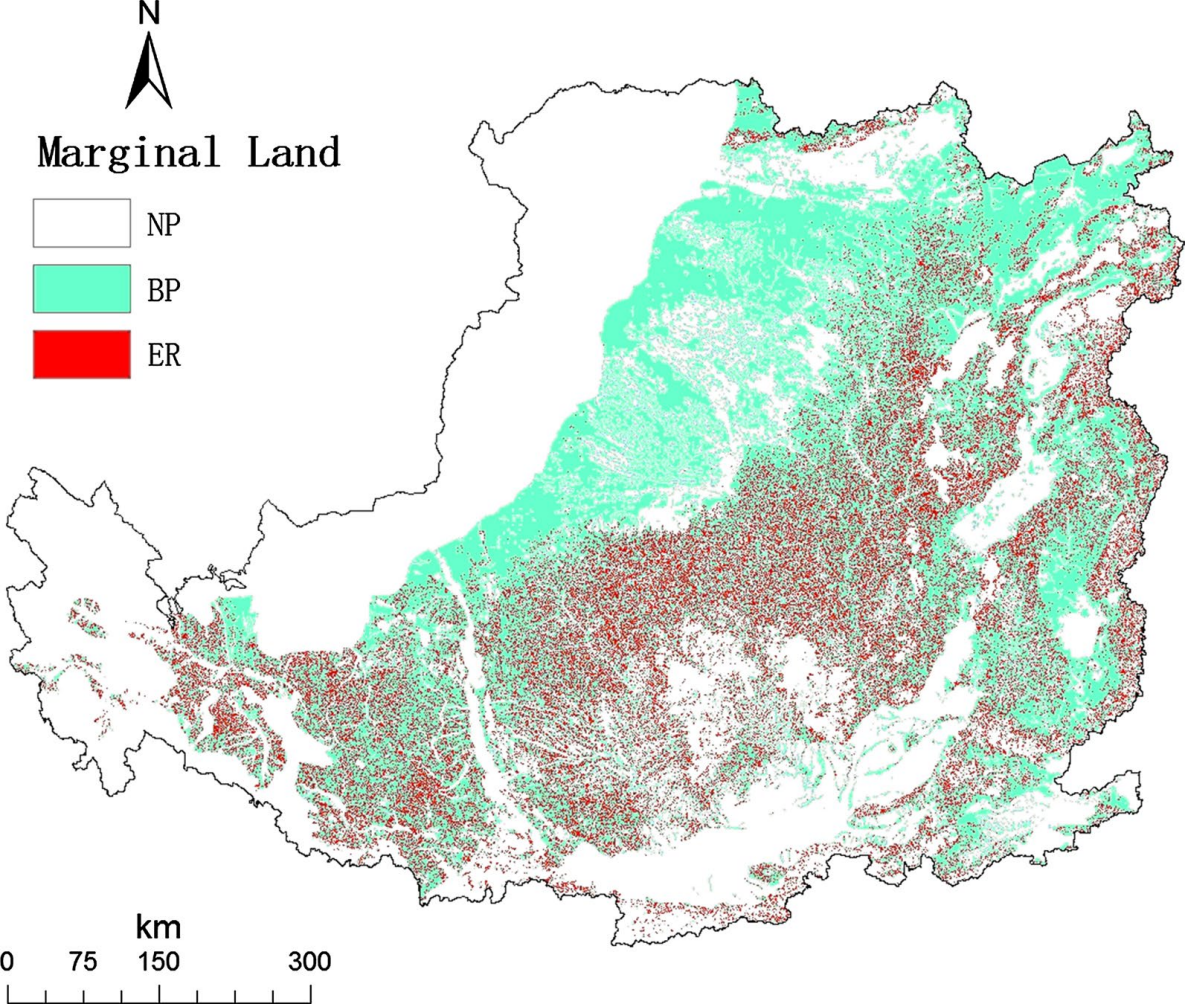
Map of suitable marginal land for planting *Miscanthus lutarioriparius* in the Loess Plateau. NP, BP, and ER indicate not suitable for planting *Miscanthus*, suitable for bioenergy production, and suitable for ecological restoration, respectively

Figure 4 showed the final map of marginal land for suitable for bioenergy production and suitable for ecological restoration using *Miscanthus* in the Loess Plateau. The result showed that there were 27.6 Mha for energy production and 9.7 Mha for ecological restoration. The northwestern and western parts of the Loess Plateau are not suitable for bioenergy production because of harsh natural conditions [32]. As for the areas lied in the southern and eastern parts are not suitable because of suitable natural conditions for crop or forest [32].

Compared to previous methods identifying marginal land [14, 18, 28, 35–38], the new index (the velocity of LUC) has some obvious advantages. First, it is generic and is not limited to a specific energy crop, which can make a direct comparison in different regions or countries. Second, it makes full use of the long-term land-use data with high resolution. Finally, it can be easily extended to larger spatial analyses, e.g., in 5 × 5 grid cell when high-performance computing is available.

**Fig. 5:**
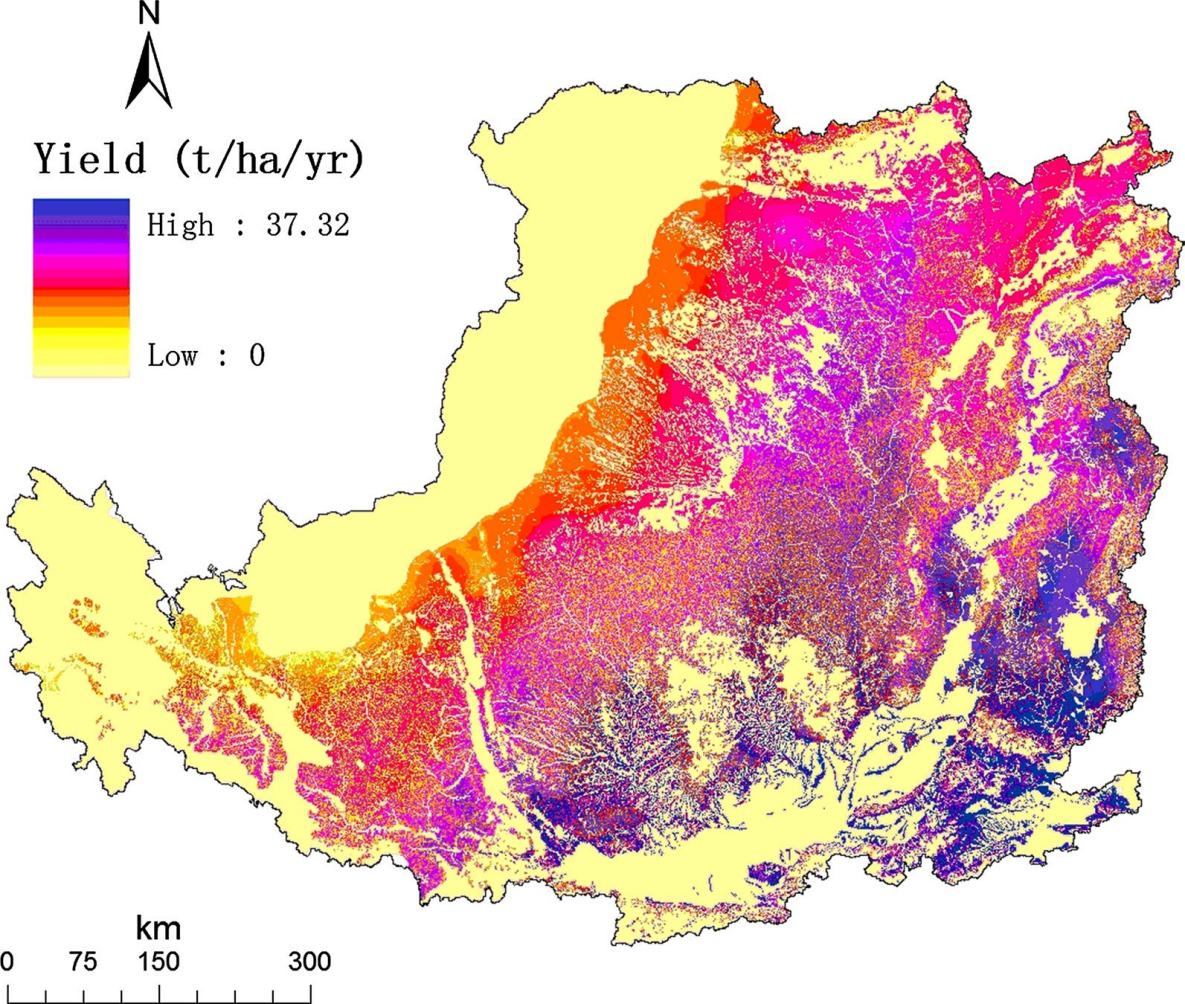
Map of the yield potential (t/ha/year) of *Miscanthus lutarioriparius* in the Loess Plateau

Based on the field experiment [29] and the previous yield model [32], we mapped the average yield potential of *M. lutarioriparius* in the Loess Plateau under three emission scenarios (A2, B2, and A1B) in the 21st century (Fig. 5). The average annual biomass production is of 0.41 billion tons and could generate ~598 TWh electricity that accounted for ~10 % of China’s electricity output in 2015. The average yield was about 15 t ha^−1^year^−1^ in the central region, which is acceptable for large-scale production and can provide a considerable amount of energy output for local development in the future.

**Fig. 6:**
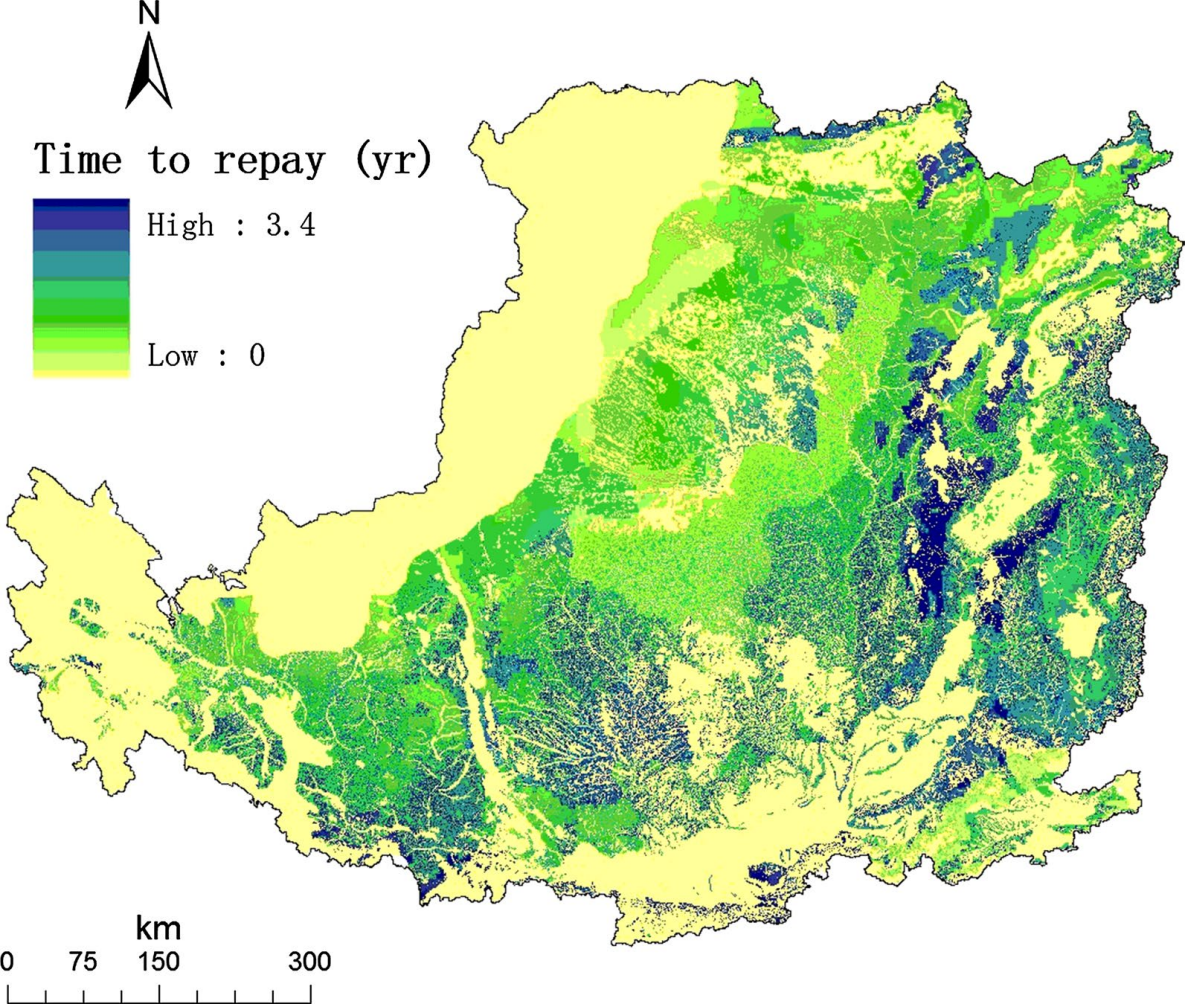
Map of the time to repay carbon debt (year) due to land-use change for planting *Miscanthus lutarioriparius* in the Loess Plateau

Furthermore, we estimated that soil carbon sequestration and total CO_2_ mitigation to be 9.3 Mt C year^−1^ and 542 Mt year^−1^, respectively. More importantly, we calculated the time to repay carbon debt due to LUC for planting *M. lutarioriparius* in the Loess Plateau (Fig. 6). The average time was 0.97 year, which implied that there was little carbon debt and short time to repay for developing bioenergy. The payback time in the Loess Plateau was similar to that in the marginal cropland planted with perennial grasses in US [4, 14] and was much shorter than that in the tropics [5, 9]. One reasonable explanation is to grow second-generation energy crops on marginal land taking advantages from both strong carbon sequestration ability of the perennial crops and low carbon stock of the marginal land.

Previous bioenergy scenario and carbon emission assessments were mainly based on the status of land use and neglected land-use history [37, 38]. In fact, land-use change was greatly affected by both environmental change and human activities, especially by economics and policy, such as Conservation Reserve Program in USA [39]. Recently, China has launched an ambitious national ecosystem restoration program called Grain to Green Program over the last decade [23]. In the coming decades after 2008, the Loess Plateau ecosystem had shifted from a net carbon source in 1980 to a net carbon sink [22]. Thus, actual LUC trajectory in the long term revealed the interaction of human activity with environmental change and may provide unique information to identifying potential marginal land.

On the other side, land use in this region is always situated in the farming-pastoral zone shifting in history due to natural and political instability [40]. For example, a large amount of cultivated land in the loess hilly areas has been idled or converted into grassland in a recent decade due to Grain for Green Project and the regional economic transition [23]. Compared with food crops, the second-generation energy crops adapt better to this more capricious environment in history, where this kind of marginal land was more sensitive to climate change and appeared rather vulnerable to environmental degradation. Growing the perennial energy crops on land with relatively poor soil conditions can restore soil fertility and improve land productivity with little carbon debt. Finally, the rotation between food and perennial energy crops may contribute to the development of a more sustainable agricultural system in regions, where land had been overused for growing annual food crops and suffered from degradation.

## Conclusions

We quantified the sustainability of *Miscanthus* planted in vast marginal land. We proposed a general approach to identify marginal land for energy crops production at large temporal–spatial scale using long-term high-resolution land-use maps. We showed that *Miscanthus* production in suitable marginal land could offer considerable renewable energy and mitigate climate change with little carbon debt. These results indicated that the Loess Plateau was suitable for *Miscanthus* production at regional scale, which provided a promising development solution to vast ecologically fragile region. In addition, the new approach tracking the land-use change history is especially suitable for those regions rich of marginal land, where it has undergone frequent land-use change due to climate change and human activities. Thus, our finding not only contributes to vast region with serious human-environment or civilization conflict, but also helps policy makers to develop better sustainable strategies to mitigate climate change.

## Methods

### The study area

The Loess Plateau is located at middle latitude and extends from central to northwestern China (34–45°N, 101–114°E) and covers an area of more than 60 Mha (Additional file 1: Figure S2). It has unique landscape, including a variety of topography, such as hilly area and plateau-gully area (Additional file 1: Figure S3). The climate is the warm or temperate continental monsoon climate with high interannual precipitation variability [41]. The region spans from arid, semiarid to semihumid zones and, therefore, is considered to be a semiarid-to-semihumid transitional zone and a farming-pastoral transitional zone. Meanwhile, it is sensitive to climate change and favors grasses over trees for restoration [42].

### Land-use type and change

Land-use data of China at the scale of 1:100,000 for 1980, 1990, 2000, and 2008 were used in this study. The data set was obtained through Landsat™ (Thhematic Mapper) and CBERS-2 (China-Brazil Earth Resources satellite) satellite images and interpreted by experts in the Data Center for Resources and Environmental Sciences, Chinese Academy of Sciences (Data Sharing Infrastructure of Earth System Science. Available from: http://www.geodata.cn/) [43]. A set of land data from field surveys was selected to guarantee the accuracy of land-use classification [44]. It was the newest land-use data set with high-spatial resolution of 30 × 30 m in China. It is regarded as the most fundamental data for the identification of marginal land which could be potentially used for the development of sustainable biomass energy.

Land-use types included six categories and 25 sub-categories, listed as follows: cropland (paddy and dry land), woodland (forest, shrub, woods, and others), grassland (dense, moderate and sparse grass), water body (stream and rivers, lakes, reservoir and ponds, permanent ice and snow, beach and shore, and bottomland), built-up land (urban area, rural settlements, and others such as roads and airports), and unused land (sandy land, Gobi, Salina, swampland, bare soil, bare rock, and others such as alpine desert and tundra).

According to the area of each land-use type, we reduced the land-use type from six to four categories, including cropland, woodland, grassland, and sandy and saline land, which cover more than 95 % of the total area in the Loess Plateau (Additional file 1: Table S2). Because *Miscanthus* may be grown on poor lands or soils with elevated salinity [45], sandy and saline land were included. We further focused on six types of all LUC types, including those from cropland to grassland, from cropland to woodland, restoration from sandy and saline, vegetation change, conversion into cropland, and conversion into sandy and saline land.

### The velocity of land-use change

For a given grid cell, its type falls into one of 25 land-use types. The temporal change from 1980 to 2008 was calculated using the number of land-use types in 4 years. Because there are only four years, including 1980, 1990, 2000, and 2008, the possible value ranged from 1 to 4. For a given grid cell, the spatial change in a given year was calculated as the number of land-use types of all nine grids in a 3 × 3 grid cell (90 × 90 m, nearly hectometer grids). The possible value ranged from 1 to 9.

Unified the two indexes, we calculated the velocity of land-use change, as the ratio of the temporal change and the average spatial change from 1980 to 2008 at a given grid [46]. The velocity of land-use change is regarded as an intrinsic character of a given grid, which reveals the evolving interaction of environmental change and human activity in the long term. Thus, we can identify marginal land for growing energy crops as the grid cell with the relatively larger temporal change and the relatively smaller spatial change.

### Identification of marginal land

We gave an operational working definition of marginal land. First, we adopted the criterion of land-use type and excluded land incapable of supporting *Miscanthus* growth, such as deserts, glaciers, and cities and high-quality cropland, pasture, and forests, such as paddy, forest, and dense grassland in 2008. Second, we adopted the climatic criterion for *Miscanthus* and excluded land which is not suitable for *Miscanthus,* such as areas, where annual precipitation is lower than 200 mm. Third, we excluded the grids with low velocity of land-use change. More details of the velocity of land-use were discussed above. Finally, land with slope over 25° was also excluded from bioenergy production and listed into the ecological restoration area, because water loss and soil erosion occurred easily for this kind of land.

### Yield model

The yield potential was calculated based on the climatic data and the yield model that was previously developed for estimating the yield potential of *Miscanthus* energy crops in the Loess Plateau based on the yield of *M. lutarioriparius* measured in an experimental field located in Qingyang, Gansu Province in China from 2009 [32]. This yield model was derived from the radiation model [47], especially with reference to its applications in the field study of *M*. ×*giganteus* [48]. In the study of *M*. *lutarioriparius*, the model incorporated the variation of growing season length described by the nonlinear relation of annual accumulated temperature over 10 °C. Water limitation reflected by precipitation was also taken into consideration given that the Loess Plateau is largely an arid and semiarid area.

To evaluate the validity of the yield model that was developed based on biomass yield in the 2010 growing season, we tested the consistence between the measured and model-predicted yield for the 2011 and 2012 growing seasons (Additional file 1: Figure S4 and S5). This observation suggested that the model can reliably predict the yield of the subsequent growing seasons. The average yields potential under three emission scenarios (A2, B2, and A1B) in the 21st century were based on our previous work on the impact of climate change on the yield in the Loess Plateau [33].

### Carbon stock and carbon debt

Given a grid cell, the time to repay carbon debt was calculated by the carbon stock before planting *Miscanthus*, the loss of soil carbon stock from one land-use type to *M*iscanthus production, and the rate of soil carbon stock accumulation in *Miscanthus* field. The time was equal to the ratio of the difference between the initial carbon stock and the loss and the accumulation rate [49]. The initial carbon stock was based on the historical LUC on each grid cell from 1980 to 2008 using bookkeeping or stock-flow method [50].

We assumed that each land-use status lasted the whole period between two remote sensing, such as 10 years between 1980 and 1990 or 8 years between 2000 and 2008. The rate of soil carbon stock accumulation in a given type of land use and the loss from one type to another was obtained from the 2006 IPCC guidelines for National Greenhouse Gas Inventories. The land-use type included grassland, cropland, woodland, and saline sand, respectively. We classified *Miscanthus* as grassland, since it is the closest land-use type based on available data. Soil carbon sequestration and total CO_2_ mitigation were estimated on the average yield from three scenarios [21].

### Parameters and data sources

An authorized terrain data set including elevation and slope at the scale of 1:100,000 were supplied by State Bureau of Surveying and Cartography of China (Data Sharing Infrastructure of Earth System Science. Available from: http://www.geodata.cn/). Data of precipitation, air temperature, and radiation, these key factors for plant growth, were obtained by China Meteorological Administration (China Meteorological Data Sharing Service System. Available from: http://cdc.cma.gov.cn/). The initial carbon stock in the soil on a given grid cell in 1980 was obtained by the database called the 1 × 1 km grid carbon stock in China (Data Sharing Infrastructure of Earth System Science. Available from: http://www.geodata.cn/). The rate of soil carbon stock accumulation in *M*iscanthus field was measured in Qingyang field [34]. All spatial analyses were conducted using ArcGIS 9.3 (ESRI Inc., Redlands, CA, USA). All simulation analyses were conducted using R 3.1.0 (R Foundation for Statistical Computing, Vienna, Austria).

## Additional file

10.1186/s13068-016-0586-y Maps of the spatial density distribution of land-use change in the Loess Plateau in three periods. **Figure S2.** Map of China showing Loess Plateau. **Figure S3.** Map of the Loess Plateau. **Figure S4.** Relationship between tiller volume and biomass of *Miscanthus lutarioriparius*.** Figure S5.** Validation of the yield model. **Table S1.** Areas of six land-use change types in three periods in the Loess Plateau. **Table S2.** Areas of each land use type in four years in the Loess Plateau.

## Abbreviations

IPCC: Intergovernmental Panel on Climate Change
LUC: Land-use change
