# Supplementary material for: Sustainable bioenergy production with little carbon debt in the Loess Plateau of China
Source: Biotechnol Biofuels. 2016 Aug 2;9:161. doi: 10.1186/s13068-016-0586-y (PMC4971626; doi:10.1186/s13068-016-0586-y)
Supplement: Supplementary file 1 — 10.1186/s13068-016-0586-y Maps of the spatial density distribution of land-use change in the Loess Plateau in three periods. Figure S2. Map of China showing Loess Plateau. Figure S3. Map of the Loess Plateau. Figure S4. Relationship between tiller volume and biomass of Miscanthus lutarioriparius. Figure S5. Validation of the yield model. Table S1. Areas of six land-use change types in three periods in the Loess Plateau. Table S2. Areas of each land use type in four years in the Loess Plateau. [file 13068_2016_586_MOESM1_ESM.pdf]

Supporting Information

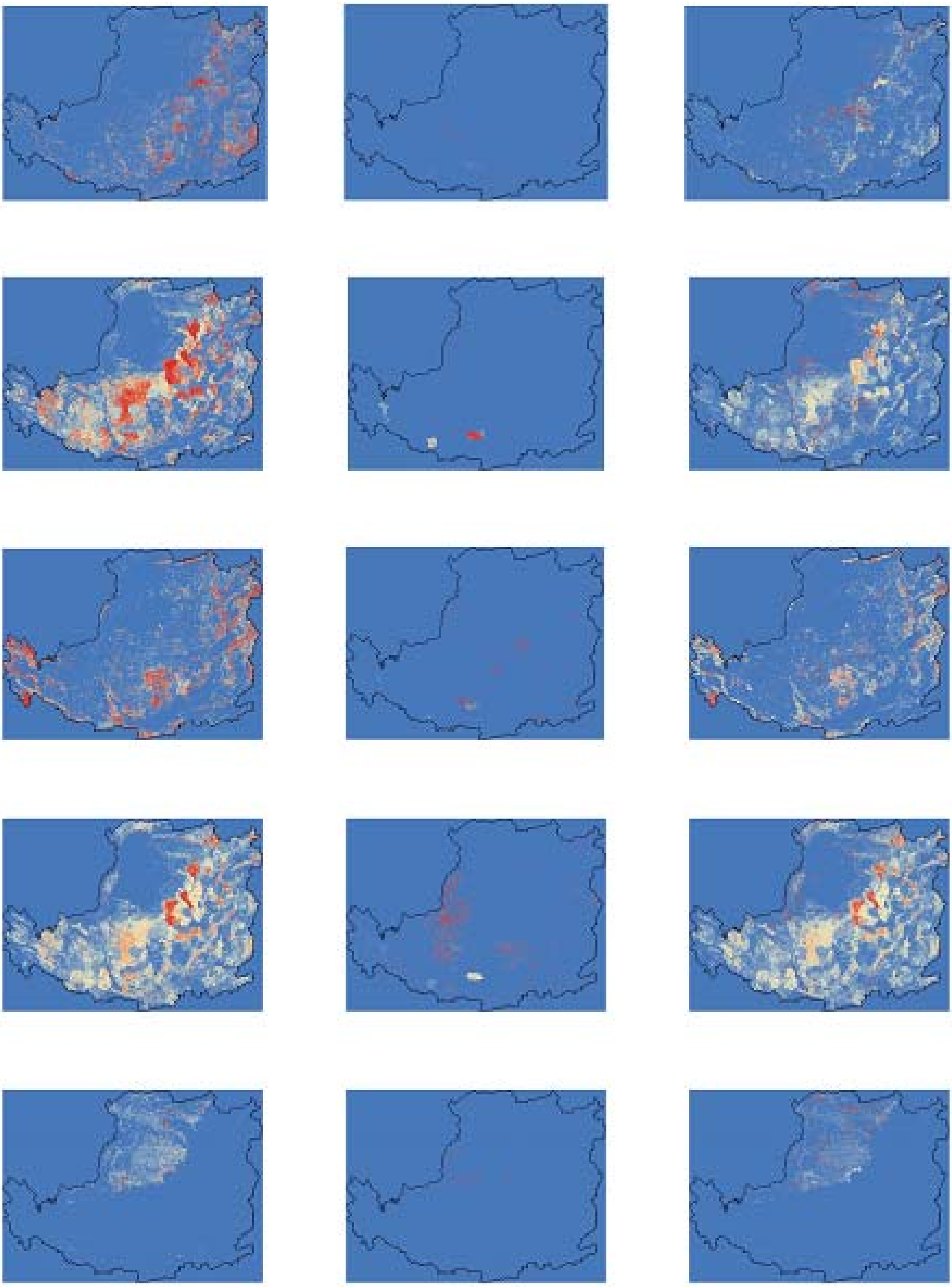

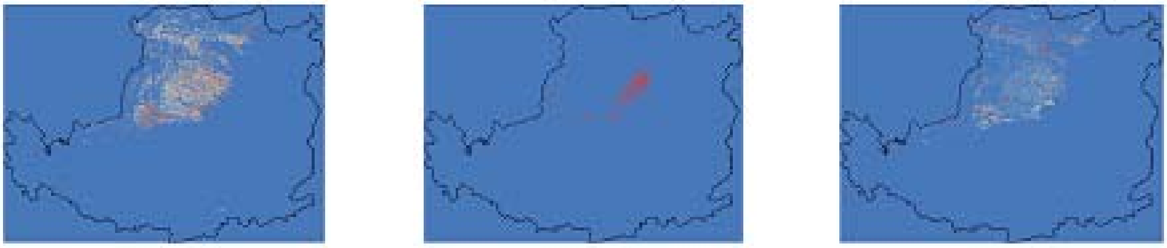

Figure S1 Maps of the spatial density distribution of land use change in the Loess Plateau in three periods. Six types of land use change from top to bottom: from cropland to woodland, from cropland to grassland, vegetation change, conversion into cropland, conversion into sandy and saline land, and restoration from sandy and saline land. Three periods from left to right: from 1980 to 1990, from 1990 to 2000, and from 2000 to 2008. Red and yellow represent high and low density; blue represents no land use change.

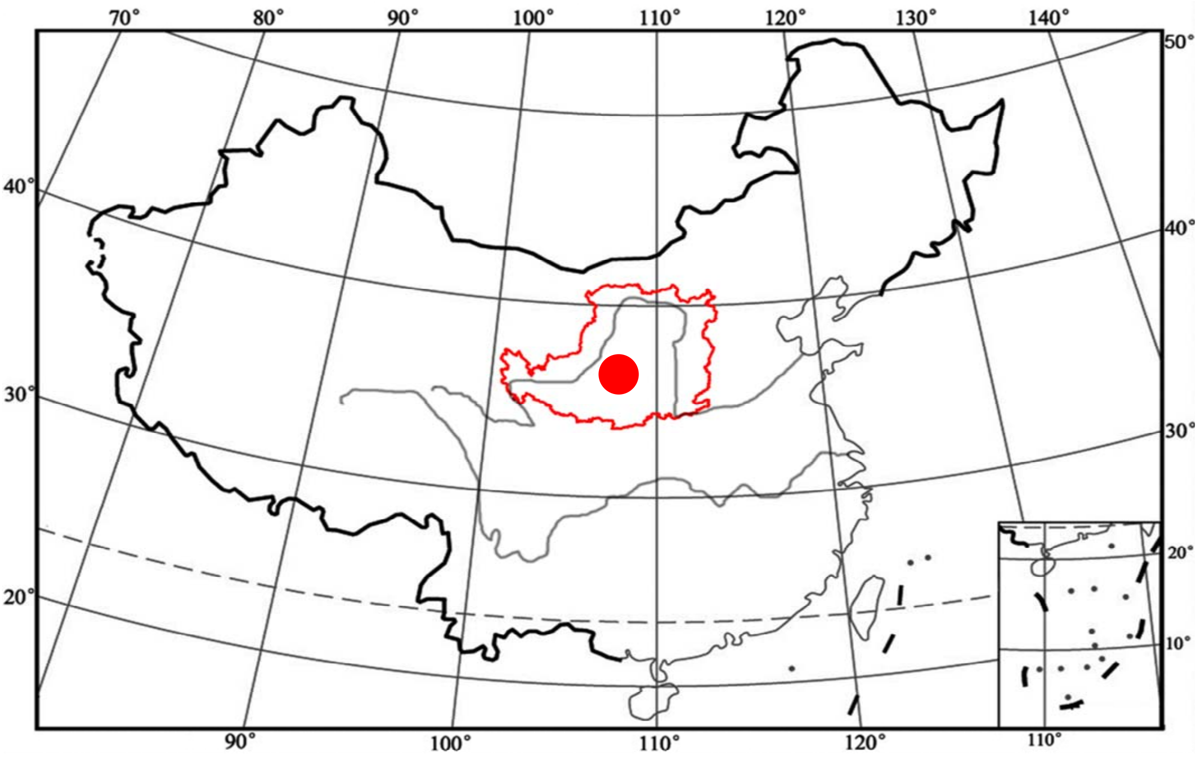

Figure S2 Map of China showing Loess Plateau. The Loess Plateau of China is outlined by the red line. Red dot represents the location of *Miscanthus* field site.

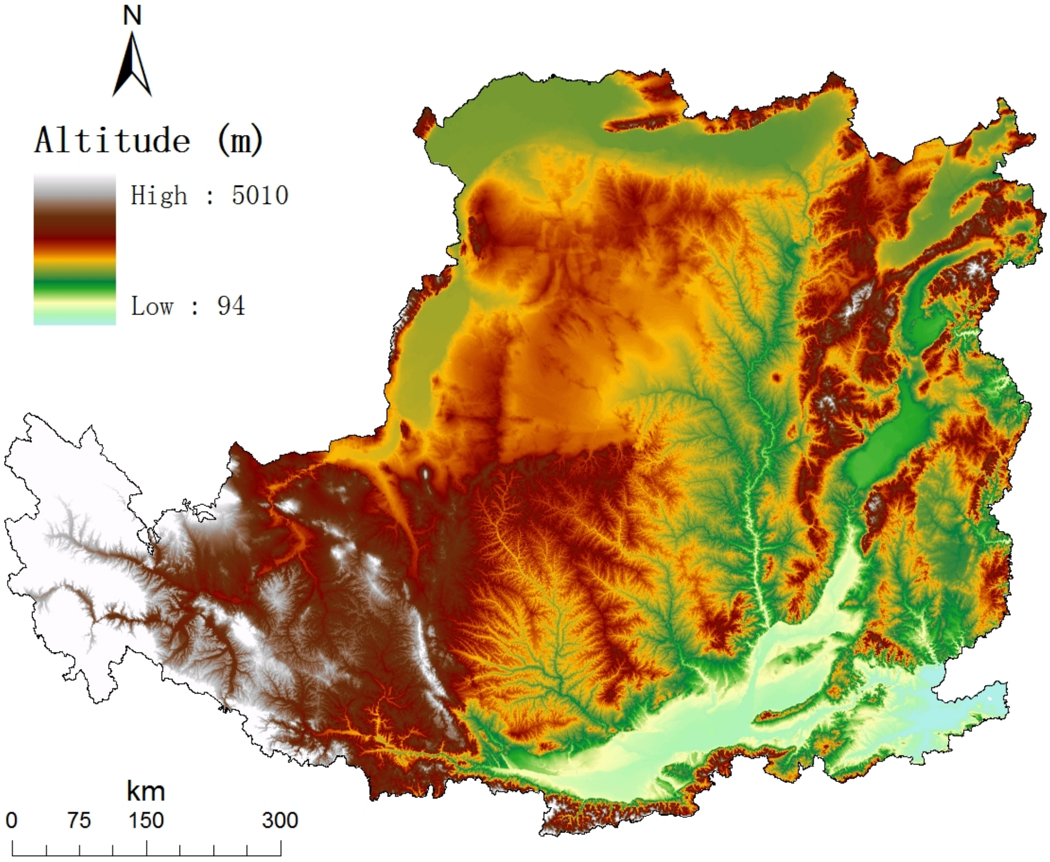

Figure S3 Map of the Loess Plateau. The Loess Plateau of China is outlined by the black line, where the average annual temperatures ranged from 0.6 to 14.5°C, the average annual precipitations ranged from 133.8 to 917.3 mm, and the altitudes ranged from 94 to 5,010 m.

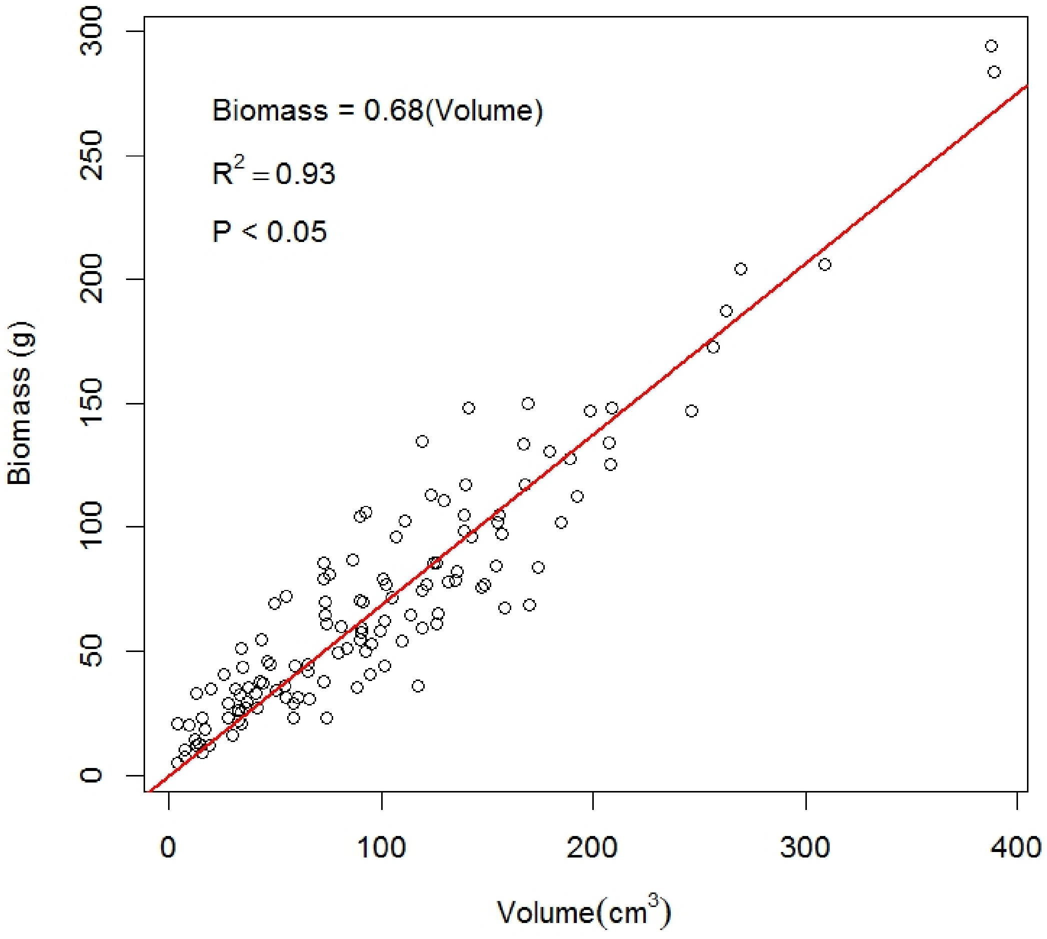

Figure S4 Relationship between tiller volume and biomass of *Miscanthus lutarioriparius*. The individuals were measured at the end of 2010 growing season in the experimental field in Qingyang of the Loess Plateau. Circles represent 112 individuals measured. Red line represents linear regression.

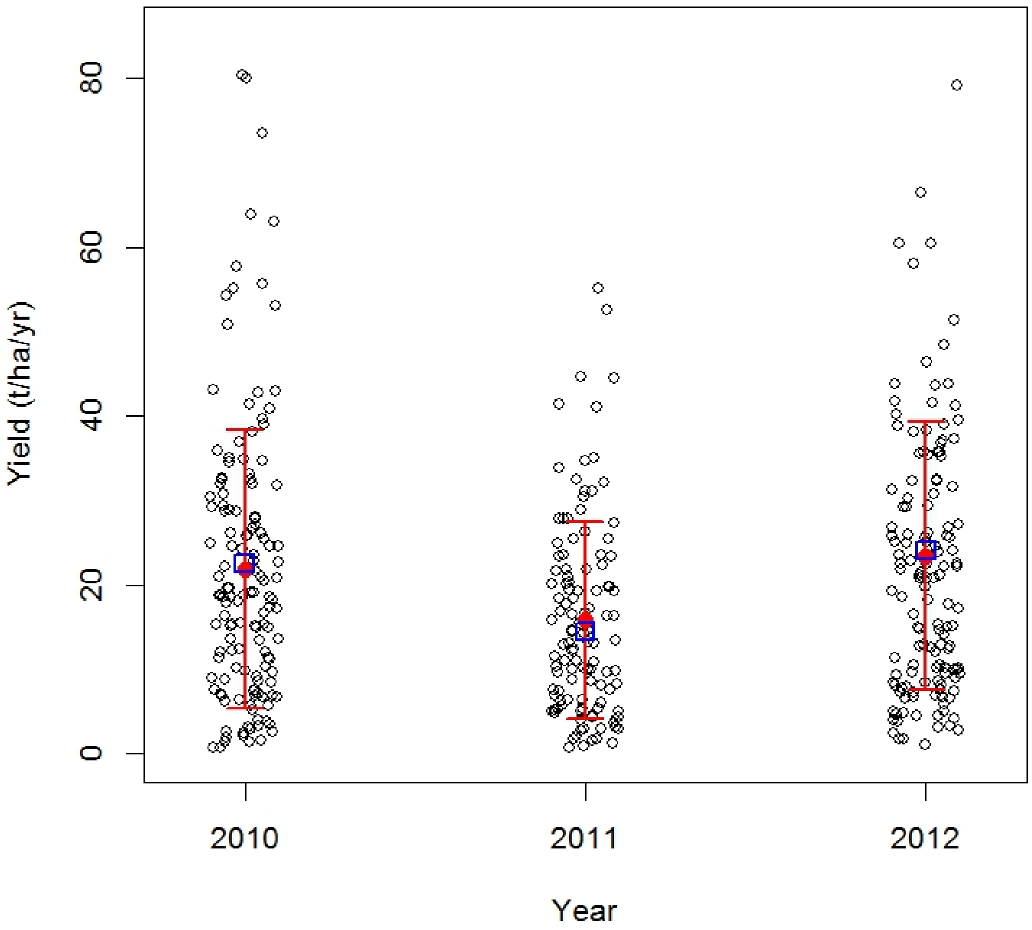

Figure S5 Validation of the yield model. Comparison of yield of *Miscanthus lutarioriparius* individuals based on field measurement and model prediction for the 2010, 2011, and 2012 growing season in the Qingyang experimental field. Circles represent field measurement for 112 individuals at the ends of the three consecutive growing seasons. The red dots and lines indicate the averages and standard deviations of the measurements. The blue squares indicate the model prediction. The *t*-test shows that the measured yield is not significantly different from the model prediction for the 2011 and 2012 growing seasons (P = 0.55, n =112; P = 0.10, n =112).

Table S1 Areas of six land use change types in three periods in the Loess Plateau.

Area (Mha)	1980-1990	1990-2000	2000-2008
From cropland to woodland	0.0842	0.0252	0.2366
From cropland to grassland	0.5059	0.0263	0.8220
Restoration from sandy and saline land	0.0371	0.1480	0.1381
Vegetation change	0.2687	0.1293	0.3771
Conversion into cropland	0.7355	0.4021	0.7702
Conversion into sandy and saline land	0.0772	0.0392	0.1863
Total	1.7085	0.7700	2.5301

Table S2 Areas of each land use type in four years in the Loess Plateau.

1st level classes	2nd level classes	1980	1990	2000	2008
Cropland	Paddy	0.54	0.54	0.60	0.59
	Dry land	19.93	19.95	20.13	19.54
Woodland	Forest	3.40	3.40	3.36	3.36
	Shrub	3.92	3.92	3.92	3.97
	Woods	1.75	1.74	1.75	1.76
	Others	0.16	0.16	0.19	0.37
Grassland	Dense Grassland	4.61	4.61	4.62	4.54
	Moderate Grassland	12.13	12.15	12.04	12.11
	Sparse Grassland	9.40	9.33	9.25	9.40
Water body	Stream and rivers	0.31	0.25	0.23	0.22
	Lakes	0.08	0.07	0.08	0.07
	reservoir and ponds	0.12	0.11	0.11	0.14
	Permanent ice and snow	0.00	0.00	0.00	0.00
	Beach and shore	0.00	0.00	0.00	0.00
	Bottomland	0.45	0.45	0.44	0.45
Built-up land	Urban area	0.18	0.20	0.25	0.32
	Rural settlements	1.08	1.09	1.14	1.20
	Others	0.08	0.09	0.10	0.15
Unused land	Sandy land	3.21	3.22	3.09	3.06
	Gobi	0.16	0.17	0.17	0.16
	Salina	0.36	0.37	0.37	0.42
	Swampland	0.08	0.09	0.09	0.08
	Bare soil	0.10	0.10	0.10	0.12
	Bare rock	0.28	0.28	0.28	0.28
	Others	0.11	0.11	0.11	0.11
Total areas (Mha)		62.41	62.41	62.41	62.41
